# Nursing home adjustment in China: mediating and moderating effects

**DOI:** 10.1186/s12877-023-03758-9

**Published:** 2023-01-27

**Authors:** Binbin Yong, Yanyan Zhang, Huimin Xiao

**Affiliations:** School of Nursing, Fujian Medical University, NO 1 Xuefu North Road, University Town, Fuzhou City, Fujian Province China

**Keywords:** Nursing home adjustment, older adults, mediating effect, moderating effect

## Abstract

**Purpose:**

Adjusting to nursing homes contributes to successful aging in older adults. However, the effect of stress on psychological adjustment in nursing home residents is unclear. Therefore, this study aimed to explore the relationship between stress and psychological adjustment among nursing home residents.

**Methods:**

This was a cross-sectional, multicenter survey. A total of 386 residents from 11 nursing homes were included in the study. Bootstrapping with resampling strategies was used to examine multiple mediators and the moderator effect. This research conforms with the STROBE checklist.

**Results:**

Path analysis demonstrated that stress directly negatively predicted the psychological adjustment of nursing home residents. It also indirectly predicted psychological adjustment through the mediating role of learned resourcefulness and self-efficacy, as well as the chain mediating role of both. Social support played a moderating role in the path of stress to learned resourcefulness.

**Conclusion:**

This study revealed the mechanism underlying the effect of stress on psychological adjustment in nursing home residents without cognitive impairment and walking difficulties. It further suggests that health providers could enhance older adults' learned resourcefulness, self-efficacy, and social support to assist them in adjusting to nursing home life.

## Introduction

The global population is rapidly aging. The number of older adults aged 60 years or older is projected to reach 2.1 billion by 2050, or 22% of the world population [1]. The demand for nursing home care continues to rise because of the increasing number of older adults with physical and cognitive function deterioration. However, moving to a nursing home marks the transition to another life stage, which is often associated with uncertainty and stress [2]. It often drastically reduces and potentially destroys family, social, and emotional attachments [3]. Many newly admitted nursing home residents experience the most stress during their first year because they fail to adapt to their new environment [4]. Higher levels of depression, anxiety, and loneliness, as well as more frequent insomnia and suicide attempts have been identified as negative consequences related to failure to adapt to nursing homes [5].

## Background

Nursing home adjustment (NHA) can be defined as an individual’s behavior and emotional response to a new place and reorienting themselves in the process [6, 7]. The transactional theory of stress and coping (TTSC) [8] presents stress as a product of a transaction between a person and a complex environment. Stress occurs when individuals think that the challenges of environmental change outweigh their coping capacity and resources [9], which may be the most important reason older adults are unable to adapt to nursing homes. Various studies have built further mediation or moderation models to explore the underlying mechanisms linking stress and NHA [6, 7, 10].

Learned resourcefulness is an adjustable intermediate variable in stress events and goal behavior [11], which can help in managing various stressful events [12–14]. It is an accumulated response model to stressful events, which involves stress-related belief-cognitive-behavioral skills to mobilize personal and social coping resources [15–17]. Learned resourcefulness plays an important role in mastering coping strategies, reducing negative emotions, and improving psychological adjustment among older adults [18, 19] and it affects self-efficacy through a variety of direct and indirect experiences [20].

Self-efficacy is defined as beliefs in one’s capacity to organize and execute the behaviors necessary to produce given attainments [21]. It is the only factor that has been consistently found to be significantly associated with NHA [10]. Nursing home residents depend on their perceived capabilities to cope with stressors of transition and mobilize the resources it needs [22]. Mateusz et al. found that high perceived self-efficacy significantly affected social integration, while low levels of self-efficacy were related to helplessness, anxiety, and depression [23].

Social support can also buffer the effect of stressful life events on older adults’ health [24, 25]. Positive social connectedness, such as support from family and peers/friends, and participation in leisure activities, can promote psychological adaptation and adaptive behaviors in living in nursing homes [5, 26]. In addition, Rosenbaum [17] points out that social support may improve an individual's resourcefulness, as most social factors can be conceptualized as indicators of social resources—the degree of active participation of family, friends, and social groups.

To further explore the factors affecting NHA, we recently completed a systematic review [10] and self-efficacy and social support, two easy-to-improve factors of high evidence, were extracted. However, existing studies have seldom explored how they influence the relationship between stress and nursing home adjustment. This is the first study to examine the association between stress, learned resourcefulness, self-efficacy, social support, and NHA, generating new insights into the mechanisms underlying the effect of stress on NHA.

## Hypotheses

**Fig. 1:**
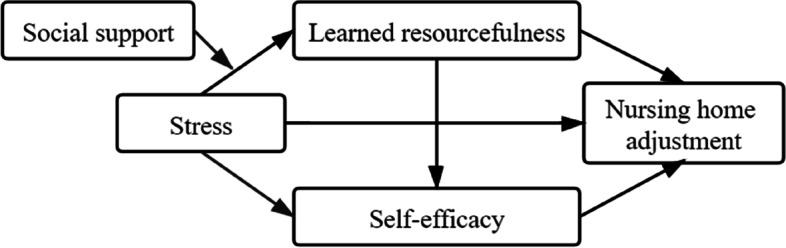
Proposed moderated mediation model. *Note:* Model fit indices: χ.^2^/df = 2, *P* < 0.001; CFI = 0.964; TLI = 0.936; RMSEA = 0.058

Based on the theoretical and empirical background, we propose the *mediation hypothesis*:

The effect of stress on NHA occurs directly and indirectly via two mediators (learned resourcefulness and self-efficacy) while controlling for confounders (see Fig. 1).

- Higher stress is associated with lower NHA among nursing home residents.
- Learned resourcefulness mediates the association between stress and NHA among nursing home residents.
- Self-efficacy mediates the association between stress and NHA among nursing home residents.
- The relationship between stress and NHA is mediated by learned resourcefulness and self-efficacy.

We also propose the following *moderation hypothesis*:

The relationship between stress and learned resourcefulness varies at different social support levels while controlling for confounders (see Fig. 1).

## Methods

### Study design and participants

This was a multicenter cross-sectional survey using cluster sampling. A total of 386 older adults were recruited from 11 nursing homes in Southeast China to participate in this study from April to August 2019. These nursing homes have all passed the national service quality evaluation based on the same requirements each year. The inclusion criteria for the participants were (i) aged 60 years or older and (ii) able to communicate in Mandarin Chinese. The exclusion criteria were as follows: (i) having cognitive impairment (Short Portable Mental Status Questionnaire, SPMSQ ≥ 3) and (ii) participation in psychosocial interventions during the study period.

### Measures

#### Demographic variables

The self-reported personal information form was designed to collect socio-demographic data, including age, gender, education level, marital status, religion, health insurance, activities of daily living (ADLs), admission decision, admission preparation, and length of stay in nursing homes. Katz Index of Independence in Activities of Daily Living Scale (Katz ADL) was used to assess ADL in the study [27]. It has six items including bathing, dressing, going to toilet, transferring, continence, and feeding. Each item is rated as 0, 1, or 2, indicating ‘dependent’, ‘partly dependent’, and ‘independent’ respectively. The total score ranges from 0 (fully dependent) to 12 (fully independent).

### Measurement tools

#### Perceived Stress Scale (PSS)

Stress, an independent variable, was measured using the PSS developed by Cohen et al. [28]. This study adopted the Chinese version of PSS (CPSS), revised by Yang et al. [29]. The scale consisted of 14 items covering two dimensions—loss of control and sense of tension—with a total of 14 items. Each item was scored on a 5-point Likert scale, and the total score was the sum of scores of all items. The higher the score, the greater the stress. The Cronbach’s α coefficient was 0.78 [29].

#### Resourcefulness Scale (RS)

Learned resourcefulness was measured using the RS. The scale was developed by Zauszniewski et al. [30] based on the self-control and help-seek resource scales. It was further translated and validated for the Chinese population by Wang [31]. The Chinese version of the RS consists of 28 items and two domains, including personal resourcefulness and social resourcefulness. Using a 6-point Likert scale, the score of each item was 0–5, and the total score was 0–140. The higher the score, the higher the resourcefulness. The Cronbach’s α coefficient was 0.885 [32].

#### General self-efficacy scale (GSES)

The GSES was used to measure the level of self-efficacy. The scale was first compiled by German psychologist Professor Ralf Schwarzer in 1981 and has been translated into many languages. The Chinese version was revised and translated by Caikang et al. [33]. It had 10 items, each item being scored on a Likert scale of 1–4. The total score was the sum of scores for all items. The higher the score, the higher the individual’s ability and self-confidence to cope with their environment. The Cronbach’s α was 0.87, test–retest reliability was 0.83, and half-time reliability was 0.82.

#### Social Science Research Solutions (SSRS)

Social support was assessed using the SSRS, compiled by X Shuiyuan [34]. It comprised 10 items and three domains: objective support, subjective support, and utilization of social support. The higher the score, the better the social support received by the individual. Its Cronbach’s α was 0.84, and the test–retest reliability was 0.92 [35].

#### Nursing Home Adjustment Scale (NHAS)

Korean scholar Lee [36] developed the NHAS. In our study, a Chinese version of the NHAS [37], consisting of 23 items and five dimensions, was used. Each item was evaluated on a 5-point Likert-type scale ranging from 1 (strongly disagree) to 5 (strongly agree). The total score was the sum of the items. The higher the total score, the higher the individual’s adaptability. The Cronbach’s α coefficient of the total scale was 0.87, and the test–retest reliability was 0.72.

### Data collection

Twenty-nine nursing homes registered in the local Civil Affairs Bureau. They have all accepted and passed the national service quality evaluation based on the same requirements each year. Among them, only 11 nursing homes agreed to voluntarily participate in this study, while 18 homes did not. The reasons for refusal included residents’ poor physical condition (*n* = 10), strict management system (*n* = 3), family members/employees requesting privacy (*n* = 3), and no interest in surveys (*n* = 2).

The eligible participants were informed about the study’s purpose and procedures, and written informed consent was obtained from each participant. The researcher collected the data face-to-face in private rooms at the nursing homes. If the participants had difficulties completing the questionnaire by themselves, the researcher read each item of the questionnaire aloud to the participants and recorded the responses verbatim. The interviews were one-on-one, each lasting 20–25 min. A total of 420 questionnaires were sent out, excluding invalid and incomplete questionnaires, and 386 residents’ data were finally included in the analysis. Thirty-four participants did not complete questionnaires for feeling boring to fill questionnaire items (*n* = 24), loss of interest in participation (*n* = 6), and family members’ visits (*n* = 4). The incidence of depression among nursing home residents was 37.49% (95% CI: 32.91% to 42.19%; [38]. Taking the maximum rate of 42.19%, considering the withdrawal of the sample, the sample size was at least 384. Thus, the actual situation met the standard. Ethics approval was obtained from the Human Ethics Committee of the author’s university prior to the study.

### Data analysis

A descriptive analysis of sample characteristics was performed using frequency (%) for categorical variables and mean with SD for continuous variables. Auxiliary analysis of some institutional variables belonging to group characteristics as covariates. Next, associations between the independent variables, dependent variables, mediators, moderators, and control variables were conducted using the Pearson correlation test. Thereafter, a path model was estimated to examine the direct effects of the relationship between stress and NHA and their indirect effects through learned resourcefulness, self-efficacy, and social support.

In the path analysis, the direct effects of the independent variable (stress) on the dependent variable (NHA) were estimated. In addition, the indirect effects of the independent variable (stress) on the dependent variable (NHA) through the mediating variables (learned resourcefulness, self-efficacy) and moderator (social support) were estimated. The total and specific indirect effects were calculated using bootstrapping with 5000 samples. The moderation hypothesis was tested using the bootstrap moderation method, calculating the conditional effect of stress on learned resourcefulness variables at different values (− 1SD, mean, + 1SD) of the moderator (social support).

The model fit indices for path analysis included the χ^2^ test, root mean square error of approximation (RMSEA) ≤ 0.08, comparative fit index (CFI) ≥ 0.95, and Tucker Lewis index (TLI) ≥ 0.95 (Kline, 2010). Descriptive analyses were conducted using IBM SPSS Statistics 25.0, and the path models were built using Mplus Version 7.0.

## Results

### Participant characteristics

The average age of the 386 participants was 83.2 years (SD = 7.00), with 76.4% aged 80 years or older. More than half of the participants were female (66.1%), had received an education below junior college (76.2%), were without a spouse (70.2%), and had a poor self-rated health status (75.6%). The vast majority had health insurance (97.7%), were completely independent in their daily lives (87.8%), and followed no religion (96.0%). More than 50% of the participants moved to nursing institutions based on their own decision (60.6%), prepared before moving in (76.9%), lived with others or a spouse (68.1%), and had resided in the nursing home for more than 12 months (72.8%; Table 1).

**Table 1:** Participant characteristics

Variable	Frequency (%)	Scores of the NHAS (Mean, SD)	t/F	*P*-value
Age (years)		83.20(7.00)	1.664	0.023
60–69	21(5.5)	74.38(17.47)		
70–79	70(18.1)	82.04(16.34)		
80–89	224(58.0)	85.56(13.16)		
≥ 90	71(18.4)	85.28(15.21)		
Gender			3.352	0.097
Male	131(33.9)	82.54(14.98)		
Female	255(66.1)	85.14(14.37)		
Education			2.889	0.022
Primary school and below	100(26.0)	80.23(14.77)		
Middle school	75(19.4)	84.24(14.26)		
High school or technical secondary school	119(30.8)	86.31(15.02)		
Junior college	34(8.8)	85.62(11.17)		
Bachelor’s degree or above	58(15.0)	86.26(14.75)		
Spouse			1.492	0.136
Yes	115(29.8)	85.97(13.86)		
No	271(70.2)	83.54(14.88)		
Religious			0.239	0.812
Yes	54(14.0)	84.70(16.23)		
No	332(96.0)	84.19(14.36)		
Health insurance			1.052	0.369
Medical insurance for urban employees	331(85.8)	84.74(14.41)		
Medical insurance for urban residents	34(8.8)	81.41(16.21)		
New rural cooperative medical insurance	12(3.1)	79.08(15.79)		
Self-paying	9(2.3)	84.56(14.18)		
ADL by Katz index		11.48(1.75)	4.849	< 0.001
12 (Completely independent)	339(87.8)	74.85(14.75)		
1–11(Partly dependent)	47(12.2)	85.57(14.12)		
Self-rated health status			1.066	0.383
Very bad	27(7.0)	80.74(18.02)		
Bad	124(32.1)	82.64(14.85)		
Ordinary	141(36.5)	85.28(13.25)		
Good	84(21.8)	85.50(13.82)		
Very good	10(2.6)	89.20(23.62)		
Admission decision			6.378	< 0.001
Oneself	234(60.6)	88.02(12.93)		
Others	152(39.4)	78.49(15.19)		
Admission preparation			5.000	< 0.001
Yes	297(76.9)	86.24(13.90)		
No	89(23.1)	77.67(15.08)		
Admission time (month)			2.314	0.076
< 3	27(7.0)	78.30(15.84)		
3–12	78(20.2)	82.85(15.31)		
12–36	126(32.6)	84.62(14.90)		
> 36	155(40.2)	85.73(13.58)		
Resident form			6.120	0.002
Alone	123(31.9)	85.01(13.77)		
Live with spouse	86(22.2)	88.29(13.46)		
Live with others	177(45.9)	81.79(15.29)		
Monthly expenses (RMB)			1.232	0.314
≤ 2000	5(1.3)	78.60(15.42)		
2001–3000	90(23.3)	84.47(17.90)		
3001–4000	140(36.3)	85.50(14.77)		
4001–5000	77(19.9)	83.97(12.29)		
> 5000	49(12.7)	84.12(11.52)		
Not clear	25(6.5)	78.92(12.00)		

Marital status without spouse includes divorced, unmarried and widowed; *ADL* Activities of daily living, *RMB* Chinese money (US$1 = RMB 6.12)

The scores of the NHAS among nursing home residents ranged from 37–115, with an average of 84.26 (SD = 14.61). The PSS scores ranged from 3–46, with an overall mean score of 23.22 (SD = 7.27). The scores of the RS ranged from 35–120, with an overall mean score of 84.91 (SD = 13.71). The GSES scores ranged from 10–40, with an overall mean score of 24.38 (SD = 7.52). The SSRS scores ranged from 14–51, with an overall mean score of 34.63 (SD = 6.49; Table 2).

**Table 2:** Scores of NHAS, CPSS, RS, GSES and SSRS (*n* = 386)

Variable	Minimum	Maximum	Mean (SD)	Number of items	Average
Total score of the NHAS	37	115	84.26(14.61)	23	3.66(0.64)
Emotional distress	2	10	7.80(1.66)	2	3.90(0.83)
Relationship development	11	35	25.28(4.98)	7	3.61(0.71)
Acceptance of new residence	6	30	22.85(4.32)	6	3.81(0.72)
Depressed mood	6	30	20.95(5.31)	6	3.49(0.89)
Feeling at home	3	10	7.39(1.57)	2	3.70(0.79)
Total score of the PSS	3	46	23.22(7.27)	14	1.66(0.52)
Total score of the RS	35	120	84.91(13.71)	28	3.03(0.49)
Personal resourcefulness	14	74	44.95(11.67)	16	2.81(0.73)
Social resourcefulness	15	59	39.96(8.62)	12	3.33(0.72)
Total score of the GSES	10	40	24.38(7.52)	10	2.43(0.75)
Total score of the SSRS	14	51	34.63(6.49)	10	3.46(0.65)
Objective support	7	25	34.63(6.49)	4	3.91(0.97)
Subjective support	3	19	11.31(3.79)	3	3.77(1.26)
Availability of social support	3	12	7.68(2.52)	3	2.56(0.84)

Higher scores of NHAS, RS, GSES, and SSRS indicate better status; higher scores of PSS indicate the worse status of stress

*NHAS* Nursing Home Adjustment Scale, *RS* Resourcefulness Scale, *GSES* General Self-efficacy Scale, *SSRS* Social Support Rate Scale, *PSS* Perceived Stress Scale, *SD* Standard deviation

### Associations among main variables

Table 3 displays the significant associations between the main variables. Among them, there was a moderate negative association between psychological adjustment and stress (*r* = 0.599, *p* < 0.05) and a moderate positive association between psychological adjustment and self-efficacy, social support, and resourcefulness (*r* = 0.573, *r* = 0.499, *r* = 0.724, *p* < 0.05).

**Table 3:** Correlations among main variables (*n* = 386)

Variable	Perceived stress	Learned resourcefulness	Self-efficacy	Social support	NHA
Perceived stress	1				
Learned resourcefulness	-0.678***	1			
Self-efficacy	-0.656***	0.628***	1		
Social support	-0.384***	0.500***	0.489***	1	
Psychological adjustment	-0.599***	0.724***	0.573***	0.499***	1

*NHAS* Nursing Home Adjustment Scale, *RS* Resourcefulness Scale, *GSES* General Self-efficacy Scale, *SSRS* Social Support Rate Scale, *PSS* Perceived Stress Scale

^***^P < 0.001

### Mediating effect of learned resourcefulness and self-efficacy

The chain-mediated model of learned resourcefulness and self-efficacy between stress and psychological adjustment fit well (χ^2^/df = 2, *p* < 0.001; CFI = 0.964; TLI = 0.936; RMSEA = 0.058). Table 4 illustrates that the total effect of stress on psychological adjustment was significant (β = -0.572; 95% CI = -0.642 to -0.504; *p* < 0.001). The direct effects accounted for 22.0% of the total effect (β = -0.126; 95% CI = -0.239 to -0.014; *p* = 0.027), and the mediating effect accounted for 78.0% of the total effect (β = -0.446; 95% CI = -0.525 to -0.368; *p* < 0.001). Within the mediating effect, the relationship between learned resourcefulness and self-efficacy was significant (β = -0.344; 95% CI = -0.415 to -0.273; *p* < 0.001; β = -0.066; 95% CI = -0.108 to -0.024; *p* < 0.001), and accounted for 77.1% and 14.8% of the mediating effect, respectively. The chain mediation of learned resourcefulness to self-efficacy was also significant and accounted for 8.1% of the total mediation effect (β = -0.036; 95% CI = -0.059 to—0.012; *p* = 0.003). The results are presented in Table 4.

**Table 4:** Mediation effects of learned resourcefulness and self-efficacy on the relationship between perceived stress and nursing home adjustment (*n* = 386)

Path	Point estimate	Standard error	Bootstrapping bias corrected 95% CI	*P*-value
**Total effect**				
Stress → psychological adjustment	-0.572	0.035	[-0.642, -0.504]	< 0.001
**Direct effect**				
Stress → psychological adjustment	-0.126	0.057	[-0.239, -0.014]	0.027
**Mediating effect**				
Total mediating effect	-0.446	0.040	[-0.525, -0.368]	< 0.001
Stress → learned resourcefulness → psychological adjustment	-0.344	0.036	[-0.415, -0.273]	< 0.001
Stress → self-efficacy → psychological adjustment	-0.066	0.021	[-0.108, -0.024]	< 0.001
Stress → learned resourcefulness → self-efficacy → psychological adjustment	-0.036	0.012	[-0.059, -0.012]	0.003

### Moderating effect of social support

**Fig. 2:**
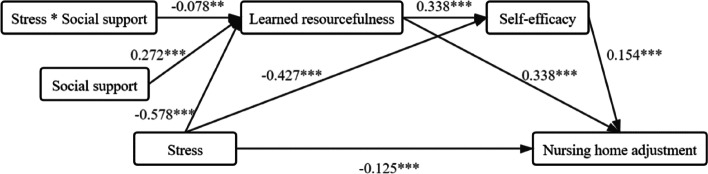
Results of final path model (*n* = 386). *Note:* (1) Model fit indices: χ^2^/df = 4, *P* < 0.001; CFI = 0.922; TLI = 0.866; RMSEA = 0.083. (2) The non-standardized coefficient is shown in the figure. ** *P* < 0.05, ***P < 0.001. (3) To simplify the model, the path coefficient of the control variable was not added

The interaction between stress and social support had a significant effect on learned resourcefulness (β = -0.078, *p* < 0.05), indicating that social support played a moderating role in the path; the model fitted well at this time (χ^2^/df = 4, *p* < 0.001; CFI = 0.922; TLI = 0.866; RMSEA = 0.083), as shown in Fig. 2. A simple slope analysis also revealed that when social support was high (higher than the mean plus the standard deviation), stress had a strong negative effect on learned resourcefulness (β = -0.656, T = -12.189, *p* < 0.001), and when it was low (less than the mean minus the standard deviation), the negative effect of stress on learned resourcefulness decreased (β = -0.500, T = -10.216, *p* < 0.001). The aforementioned results indicate that social support not only affects learned resourcefulness but also further affects psychological adjustment via the path of learned resourcefulness to self-efficacy, as detailed in Table 5.

**Table 5:** Moderating effect of social support (*n* = 386)

Moderating variable	Indirect effect size	Boot standard error	Bootstrapping bias corrected 95% CI
High-level social support	-0.197	0.101	[-0.481, -0.056]
Moderate social support	-0.171	0.086	[-0.415, -0.050]
Low-level social support	-0.145	0.071	[-0.347, -0.044]

The path by which moderating variables act: stress → learned resourcefulness → self-efficacy → psychological adjustment

## Discussion

This study found that stress was associated with NHA through the following mechanisms: (1) indirectly through learned resourcefulness, (2) indirectly through both learned resourcefulness and self-efficacy, and (3) indirectly through self-efficacy. Three specific indirect effects were found to be statistically significant. Moreover, the relationship between stress and learned resourcefulness was moderated by social support.

The results of the association analysis in this study demonstrated that there was a significant negative association between stress and psychological adjustment. The path model also showed that stress could negatively affect psychological adjustment. In other words, the greater the stress, the lower the psychological adjustment of older adults. Perceived stress is generated by the mismatch between perceived situational demands and perceived personal coping resources [8]. Therefore, stress experiences and coping results have immediate effects, such as emotional or physiological changes, as well as long-term results concerning psychological well-being, somatic health, and social functioning [8, 39]. When older adults leave their families and move to a nursing home, their personal and social coping resources are weakened, which can intensify the negative effects of stress on psychological adjustment.

Regarding self-efficacy, the association analysis of the present study indicated that self-efficacy was negatively correlated with stress and positively correlated with NHA, confirming previous research [40]. This provides a precondition for conducting the mediation analysis. According to the cognitive-relational theory of stress, general self-efficacy is considered to represent a personal resource among other antecedents of appraisals [39]. It reflects the belief in one’s ability to master challenging demands by employing adaptive action [39]. High self-efficacy buffers the experience of stress, whereas low self-efficacy puts individuals at risk for a dramatic increase in threat and loss appraisals [41]. Therefore, low self-efficacy is associated with depression, anxiety, and helplessness, and high self-efficacy increases one’s motivation to perform more challenging tasks. It is necessary to improve the self-efficacy of older adults in taking adaptive actions to alleviate the stress of resettlement and promote psychological adjustment.

Learned resourcefulness was also found to be negatively correlated with stress and positively correlated with psychological adjustment, which is consistent with results of previous studies [42, 43]. Perceived stress is determined simultaneously by perceiving environmental demands and personal resources, which can change due to coping effectiveness, altered requirements, or improvements in personal abilities [39]. Learned resourcefulness can improve one’s ability to cope [44] and can therefore buffer against perceived stress. Studies [8, 45, 46] have suggested that individuals will assess their capacity, social support, material, and other resources to adapt and re-establish the balance between themselves and their environment. Learned resourcefulness involves the assessment and use of adaptive resources [47] and, therefore, contributes to psychological adjustment.

Stress can affect psychological adjustment via the path of learned resourcefulness to self-efficacy. The results of the present study verify previous research in this regard [14, 44, 48]. Perceived self-efficacy pertains explicitly to one's coping resources [41]. This sense of competence can be acquired through mastery experience, vicarious experience, verbal persuasion, or physiological feedback [49]. Learned resourcefulness is a collection of coping strategies and skills that help individuals solve problems effectively [50]. Therefore, learned resourcefulness helps enhance self-efficacy, which can improve psychological adjustment. In contrast, if the individual underestimates his or her action potential, no adaptive strategies will be developed [49, 51]. In summary, older adults benefit more from improving their learned resourcefulness than simply improving their self-efficacy.

Concerning the role of the moderator, the results of the present study demonstrate that social support moderates the influence of stress on learned resourcefulness. Several studies have illustrated that the better the social support, the better the psychological adjustment of nursing home residents [5, 26]. Similarly, the buffer model of social support theory [25, 52] holds that individuals can buffer the negative impact of stress events through social support systems to improve social adaptation. Moreover, the learned resourcefulness theory [30] suggests that individuals can not only adopt self-help strategies but also actively seek outside professional or non-professional help. Thus, it is clear that social support contributes to coping with stress and achieving psychological adjustment.

### Limitations

This study has some limitations. First, this study had a cross-sectional design, which could not test the trajectory of NHA over time in older adults. Second, the sample was limited to those residents without cognitive impairment and walking difficulties, therefore sample selection bias existed. Third, we cannot exclude the possibility of unobserved selection bias between participants and non-participants. The future study could pay more attention on potential selection bias. Fourth, self-reported measurements may be subjective, which may result in self-report bias. Moreover, other confounding factors have not yet been considered. Future research should consider this issue more comprehensively.

## Conclusion

This is the first study to explore the nursing home adjustment path model in China for residents without cognitive impairment and walking difficulties. The results suggest that stress can directly negatively predict psychological adjustment, and it can also predict psychological adjustment indirectly through three paths: (1) the mediating role of learned resourcefulness, (2) the mediating role of self-efficacy, and (3) the chain mediating role of learned resourcefulness and self-efficacy. In addition, social support plays a moderating role in the path between stress and learned resourcefulness.

### Relevance to clinical practice

This study provides health providers with new insights into the impact of stress on nursing home adjustment. It also implies that to improve psychological adjustment, additional focus should be placed on enhancing learned resourcefulness, self-efficacy, and social support among nursing home residents. Therefore, health providers should consider a multifaceted approach to enhance the learned resourcefulness and self-efficacy of older adults and create a more positive social support atmosphere. Specifically, health providers should provide nursing home residents with a toolbox of stress coping strategies, encourage them to apply rich life experience to solve problems, and establish a positive peer-family support network. Making full use of existing resources could facilitate residents to cope with adjustment difficulties and promote active ageing.

## Data Availability

The data that support the findings of this study are available from the corresponding author upon request.
